# Enhancing Description and Interpretation of Qualitative Interviews With People With Intellectual Disabilities Through Nonverbal and Paraverbal Data Collection and Analysis

**DOI:** 10.1111/jar.70183

**Published:** 2026-01-18

**Authors:** Lynette Harper, Rob Burton, Ian Walshe, Ann Ooms

**Affiliations:** ^1^ Faculty of Health and Life Science Northumbria University Newcastle UK; ^2^ Faculty of Health, Science, Social Care and Education Kingston University London UK

**Keywords:** data collection, intellectual disabilities, interviews, learning disabilities, nonverbal data, paraverbal data, triangulation

## Abstract

**Background:**

Qualitative research involving interviews typically includes transcribing verbal data. However, insights about meaning can also be ascertained from nonverbal and paraverbal communications. Transcribing nonverbal data allows researchers to include and analyze this additional data whilst ensuring participants' confidentiality.

**Methods:**

Six participants with intellectual disabilities were interviewed using Talking Mats as a communication tool to support data collection. The verbal, nonverbal, and paraverbal data were transcribed using a notation system and analysed using triangulation.

**Findings:**

Most of the nonverbal communications corroborated the spoken word; however, nonverbal and paraverbal communication also captured additional information, which added depth, shared understanding, and expanded the insights into the research process or refuted the spoken word, which in turn provided new insights.

**Conclusions:**

This paper presents a method to analyse verbal, nonverbal and paraverbal data to provide depth and new or more accurate meaning and highlights benefits of including nonverbal communication in research.

## Lay Summary

- As well as speech, body movements, facial expressions and the way that something is said (e.g., tone, pace and pauses) can provide important information about how people with intellectual disabilities feel about their past experiences and preferences for their future.
- Symbols and brief descriptions can be used to capture nonverbal communications.
- When nonverbal communication and the way something is said is merged with what is said, it can make data collected for research richer, more accurate, and more meaningful, thereby allowing the findings to accurately reflect the participant's experiences.

## 1 Introduction

The objective of this paper is to present a method for capturing, analysing and interpreting the verbal, nonverbal and paraverbal communications obtained during interviews. Nonverbal data includes hand and body movements, gestures and positions, facial expressions, touch and use of objects. Paraverbal data includes tone, pitch, intonation, pauses and hesitations. A systematic approach was sought to enable a more comprehensive reflection on meaning gleaned from these data sources to support researchers' interpretations of participants' lived experiences. This paper does not report on the findings of the study used as an exemplar but focuses on the model used to incorporate the verbal, nonverbal and paraverbal data. The methods used to analyse the pictorial data are presented in Harper et al. (2025).

Qualitative research using interviews as data collection methods has arguably brought aspects of “the unknown about people with intellectual disabilities into the known” (Beail and Williams 2014, 93). Qualitative interviews are typically converted into transcriptions from audio recordings to understand perspectives and experiences of a particular phenomenon (Polgar and Thomas 2020). However, we argue that in addition to the spoken word, analysing nonverbal communication can aid understanding and add a powerful dimension to meaning making along with verbal interactions. Mehrabian (1971), who is frequently cited in the literature as depicting a communication model where only 7% of communication is verbal and the remaining 93% is made up of nonverbal and paraverbal communication, argues that to ensure accurate communication we need to pay attention to the overlooked contribution of nonverbal communication, which is inseparable from our feelings and projected during our interactions. We consider both nonverbal and paraverbal data. Capturing and including all formats of communication contributes to the credibility of the findings and adds additional depth and insight to the research (Onwuegbuzie et al. 2010). However, there are limited methods available on how to transcribe and analyse nonverbal interactions in interviews for the purpose of capturing information about the phenomenon under investigation, rather than the dynamics of the social interaction or the way that language is used to achieve mutual understanding.

Analysing nonverbal communication is particularly important when conducting research about participants who may have difficulties with expressing themselves verbally. Word selection, breadth of vocabulary, understanding interviewers' instructions and health literacy can present barriers to communicating lived experience and perspectives, especially for people with intellectual disabilities, people with aphasia associated with stroke or dementia, or infants who have yet to acquire the full systems of language and vocabulary breadth (Nocivelli et al. 2023; Dalemans et al. 2009). Observing nonverbal communication has been found to be useful to aid communication partners in the understanding of meaning in people with communication barriers (Bender et al. 2022). Therefore, adjusting research methods that consider all forms of communication is necessary to capture meanings of nonverbal communication.

Most research textbooks and papers focus on analysing the verbal data with only ad hoc and brief mentions of using nonverbal data from reflexive memoing or notes taken during or following interviews. Onwuegbuzie et al. (2010) and Onwuegbuzie and Byers (2014) suggest this as a substantive reason that novice researchers do not incorporate nonverbal data in their data collection, data analysis or data interpretation. However, in this study it was particularly important to include this data given that participants' levels of comprehension and expression varied.

### 1.1 Methodological Challenges in Research With People With Intellectual Disabilities

Although it is acknowledged that it would be beneficial to include nonverbal communication in research involving different groups of people, this paper focuses on people with intellectual disabilities. Researchers have previously questioned the extent to which interviewing people with intellectual disabilities can provide rich description and depth of insight relating to the topic of interest (Sigstad and Garrels 2018). Difficulties obtaining a rich data set are due to the practical challenges of recruiting larger sample sizes, obtaining depth of data from individual participants, and including people with a range of abilities (Van der Weele and Bredewold 2021; Nicholson et al. 2013). Therefore, research relating to the health and well‐being of people with intellectual disabilities often relies on proxy reports from healthcare providers or families or objective measures (Harper et al. 2023; Wolton et al. 2022). However, proxy reports are not viewed as valid substitutes for self‐reports, given the poor agreement found between the two. In contrast, person‐centred frameworks which place the person at the centre of the experience (Sigstad and Garrels 2018; Corby et al. 2015) are seen to be a more accurate measure. It is also acknowledged that people with intellectual disabilities should be more actively involved in a wider array of research and that this will require more innovative and novel approaches to methodological designs, methods of data collection and interpretation of data (Kaley et al. 2019).

Interview methods offer an opportunity to collect subjective perspectives and facilitate agency of participants with intellectual disabilities who have some verbal communication skills (Sigstad and Garrels 2018). Semi‐structured interviews are widely used to collect qualitative data but pose challenges to the researcher focusing on hearing directly encountered experiences of people with intellectual disabilities. Low response rates and limited verbal responses frequently prevent saturation within an interview and across participants, thereby only partially representing participants' views and experiences (Nelson et al. 2013; Kaley et al. 2019; Hollomotz 2018; McFarland et al. 2024). To counteract this methodological weakness, nonverbal data adds depth and clarity, allowing researchers to interpret and uncover latent meanings. Additional data captured by video recording can be used alongside other approaches, as suggested by Sigstad and Garrels (2018), including the use of prompts, rephrasing questions, and paraphrasing responses to encourage further exploration and summarising data.

A limitation of interpreting nonverbal communication is that it can be subjective and carries a risk of bias. People with intellectual disabilities may exhibit differences in nonverbal communication, which can increase the risk of misinterpretation. However, adopters of critical dialogism assert that all forms of communication are relational and carry a degree of uncertainty which leads to multiple potential interpretations (Teachman et al. 2018). Voice is therefore co‐constructed between people in any given context, which incorporates what is said before and what is expected to follow and requires us to be attuned to our embodied responses to note what is communicated through what is spoken, what is not said, and what is observed nonverbally (Broomfield et al. 2023).

### 1.2 Data Collection and Capture

Interviews with participants with intellectual disabilities require an adaptive approach tailored to individual communication needs (McFarland et al. 2024). Adaptations include visual tools or information that enhance understanding, such as easy to read participant information sheets and interviews incorporating the use of photographs, sorting of visual cards, and participant‐created artistic expressions (Schwartz et al. 2020).

This study used Talking Mats as a framework to support the first interviews with all participants. This symbol‐based communication tool supports people with communication difficulties to express their perspectives and has previously been used in a range of settings with people who have intellectual disabilities, dementia, aphasia, developmental disabilities, stroke and acquired brain injury (Cameron and Murphy 2002; Hayden et al. 2023). Talking Mats support neutral interviewing by shifting the power balance and encouraging open questioning to gain a subjective picture of a person's experience (Darvell and Bradshaw 2023). Each Talking Mat includes a topic card representing the focus of the research question, a visual top scale (thumbs up, thumbs down and so‐so) and option cards. Option cards are handed individually for participants to position according to their feelings. The visual images support shared attention and present information in manageable chunks, which encourages consideration of different areas of the phenomenon. An important component of using Talking Mats is the observation of multimodal communication, including placements of the symbols, verbal cues and nonverbal cues (Hayden et al. 2024), which all can be regarded as data and thus included in the analysis.

Video recordings enable the capture of nonverbal and paraverbal data. The use of video to capture nonverbal and paraverbal methods of expressions during interviews can support those who prefer alternative or augmentative forms of communication, which correspondingly are used instead of or to add to the spoken word. However, such recordings may not fully capture the integrated nature of verbal, paraverbal, and nonverbal communication, as fixed microphone and camera positions can miss subtle cues, broader contextual dynamics, and technical inconsistencies caused by minor shifts in participants' proximity to the equipment. In comparison, audio recording and interviewers' notes do not offer opportunities to review and reflect on the nonverbal and paraverbal communications in the same depth. Video‐recorded nonverbal communications are rarely incorporated systematically in research using interviews with people with or without intellectual disabilities (Onwuegbuzie and Byers 2014). Correspondingly, nonverbal and paraverbal data used to support the spoken word are often observed in interviews but not recorded nor time stamped for later analysis (Vanover et al. 2022). The limited research which incorporates some commentary on nonverbal interactions often excludes this nonverbal data from analysis and interpretation in a systematic and methodical way (Onwuegbuzie and Byers 2014).

### 1.3 Transcription and Analysis of Nonverbal and Paraverbal Communication

Transcribing interview data practices are subjective and can be undertaken in different ways resulting in either “thick” or “thin” transcripts (Miles and Huberman 1994; Miles et al. 2020). Denaturalism, which removes the description of nonverbal and paraverbal communication modes, produces thin transcripts, while a naturalism mode, which includes utterances, gestures, emotions, and the way things are said, is recorded to produce thick transcripts that capture the depth of the conversation (Point and Baruch 2023). Thick transcriptions can help researchers immerse themselves in the data, resulting in a better comprehension of the participants' experiences or feelings, even more so when the interviewer is involved in transcribing and thematically analysing the data (Vanover et al. 2022; Point and Baruch 2023). However, thick transcriptions are time consuming, increase the risk of error and subjectivity by deciding what to include or exclude because it is impossible to gain meaning while focusing on every nonverbal, paraverbal, and verbal element of the interview separately.

Nonverbal communication can be classified using different dimensions and therefore using a range of taxonomies. One such approach is to classify nonverbal interactions according to the part of the body that moves (Abner et al. 2015). This approach tracks the frequency of movements (e.g., head nods, hand gestures) but fails to capture information about the function of the nonverbal communication. Therefore, in this study the use of notation identifying the part of the body used to communicate was also supported by commentary explaining what was observed by the transcriber. Gorden's (1980) typology of nonverbal communication considers kinesics (body movement and posture), proxemics to indicate spatial relationships, chronemics (pauses, rhythm and hesitations in speech), paralinguistics (pitch, tone and volume to vocally express meaning). Gordon's typology highlights the need to include paraverbal as well as nonverbal communication in notation systems used for transcribing, in order to orientate the reader to useful information that can enhance their understanding. McNeill (1992) typology of nonverbal communication includes body movements that depict concrete imagery (iconic gestures) and metaphoric or abstract gestures alongside conventional gestures, including head nods or thumbs up. McNeill's (1992) typology of gestures includes a classification of iconics (simulating movements in the interview space or visually portraying an object), metaphorics (to characterise abstract perceptions and concepts), beats (e.g., movement of the hand or finger to indicate importance or timing), deictics (e.g., abstract pointing towards concepts previously discussed) and emblems that have cultural meaning.

The following sections present our methodology for capturing, analysing and interpreting the verbal, nonverbal and paraverbal data. The preparation of the data for analysis and triangulation of the verbal and nonverbal data is illustrated using excerpts from a study on sleep in people with intellectual disabilities. This provides researchers with a coherent explanation and illustrates a typology that can be adapted in future research. To provide some context to the approach taken, the study is briefly described alongside the ethical considerations taken to ensure the confidentiality of the research participants.

## 2 Method

### 2.1 Research Design

The authors of this paper conducted a research study that aimed to understand the lived experiences and preferences of people with intellectual disabilities who experience self‐reported sleep problems. To make the research more inclusive and therefore the findings more applicable, it was deemed important to invite people who use alternative and augmentative communication systems to participate. To capture different types of communication, data collection included videos of interviews with people with an intellectual disability. This generated several formats of data, namely (1) verbal data, (2) nonverbal and paraverbal data, and (3) visual data from arrangement of pictures from the Talking Mats.

To understand the individual's interpretation of the problem called for the application of innovative and dynamic ways to use frameworks, methods or tenets from multiple research methodologies. A qualitative multi‐method study was used incorporating interviews using Talking Mats and sequentially phenomenologically informed interviews (M. Van Manen 2015, 2016), with recognition of the intersubjective dimensions inherent in both waves of the research. One potential influence viewed as having both positive and negative impacts on the interpretation of the findings included personal bias. The author has more than 20 years of professional experience working with individuals with intellectual disabilities in England, which has fostered a nuanced understanding of the cultural norms, values, social expectations and unique nonverbal communication styles present in this context. However, these same previous experiences can lead to pre‐existing biases, assumptions and beliefs that may distort interpretations. To address this, a process of reflexivity was employed, recognising that individuals may communicate in ways that do not align with normative expectations, and incorporating consideration of both emic (insider) and etic (outsider) perspectives to critically examine and contextualise the interpretations. Multiple data sets (verbal and nonverbal data) were concurrently collected and analysed in strands of the study. Nonverbal data was triangulated with the verbal data using the following four codes: (1) exploring corroboration, (2) capturing additional information, (3) discovering new insight, or finally, (4) to broaden and expand insight.

### 2.2 Ethics

Prior to commencement of recruitment in 2023, ethical approval was granted from the Health Research Authority and the University Ethics Committee which included permission for video recording (reference 40069 and 22/LO/0154). Contact was made with the organisations involved who received information sheets, an easy read leaflet about the study as well as a video describing the purpose of the study and participants' rights for people who struggle with the written word. Potential participants were asked to view the materials prior to deciding if they would like to learn more about the study and meet with the research team. On the easy read consent form, it was reiterated to participants that their participation was voluntary and that they could stop the interview at any time.

### 2.3 Participants

Participants were recruited through local charity and advocacy organisations. All participants were adults living in England, verbally able to communicate their responses and to confirm verbally and through indicating on a consent form that they agreed to participate in the study, able to demonstrate abstract thinking, position cards on the Talking Mat, and had self‐reported sleep problems. Symbolic understanding, demonstrating abstract thinking and the ability to interact with the outside world is needed to use Talking Mats (Murphy et al. 2013).

Six participants agreed to participate and have the interviews recorded. Through participant information sheets, participants were informed that they could have a carer or a family member present if they wished. Two of the participants were supported by carers, and all participants had spoken to their families or support groups about involvement in the study.

The six participants (3 male and 3 female) completed two or three interviews, depending on whether the person required additional time to explain their sleep problem or to accommodate differences in attention span. All participants were aged between 18 and 65 and had a range of sleep problems, including sleep disordered breathing, experience of distressing hypnogogic states, and disrupted sleep due to a frequent need to use the toilet. In addition, some of the participants had physical health, mental health, and bereavement that impacted their sleep.

### 2.4 Equipment

**FIGURE 1:**
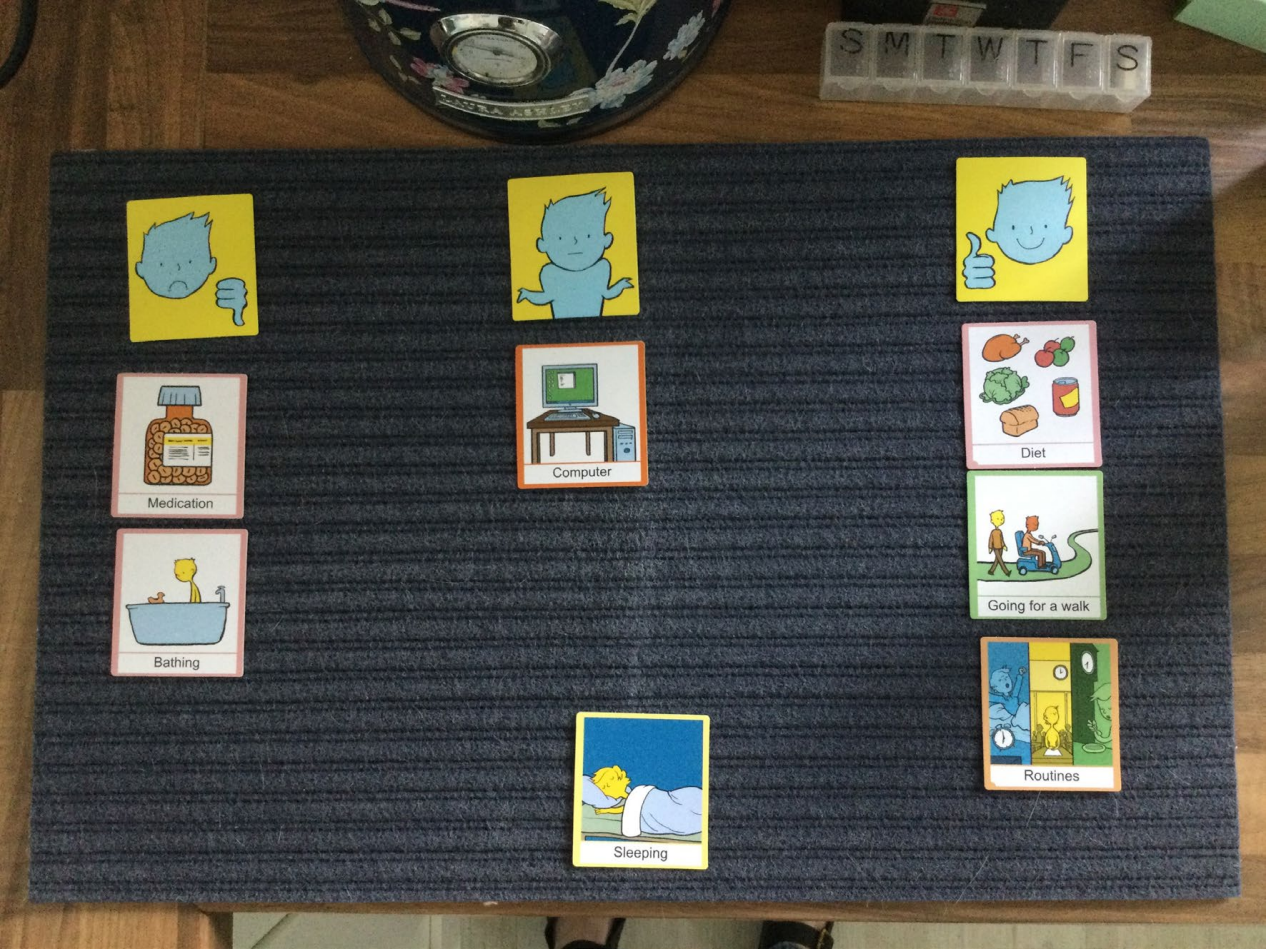
Image of a talking mat.

Four Talking Mats focusing on different aspects of sleep problems were used to facilitate interactions during a one‐to‐one, face‐to‐face semi‐structured interview with the participants. The first mat focused on what participants felt was or was not a problem for them. While waking in the night, waking early or not getting to sleep till late, and so forth, could be seen as a problem, it was more important for the study to understand if this was perceived as a problem by the individual. The second Talking Mat focused on how participants felt after a bad night's sleep and considered the self‐reported impact on their cognitive ability, emotional regulation and daily functioning. The third Talking Mat focused on causes of a bad night's sleep and the final Talking Mat focused on the strategies they would be willing to try to see if they improved their sleep (see Figure 1). A base card was used to identify that the discussion related to aspects of sleep and participants were asked an overall question about how they felt for each of the Talking Mats. Participants were given three answer options, namely, to place the picture under the thumbs up, which represented not a problem or that they were willing to try, to place under a thumbs down to show that it is a problem for them and they are not willing to try, or to place the picture in the so‐so section, meaning that they are unsure. Given the complexity of the topic, open questions and paraphrasing were used to encourage depth of data.

Visual prompt cards included known causes of disturbed sleep as well as impacts and strategies that have previously been reported in the literature as supporting sleep quality. Additional blank cards were used to encourage participants to provide additional information that was not visually depicted in the preprepared cards. For example, for sleep strategies, participants gave examples and discussed use of autonomous sensory meridian response (ASMR), specific pillows, black out blinds, and moon lamps, all of which were then categorised as strategies that they would or would not be willing to use again in the future. The video recordings capturing verbal and nonverbal data also captured images of the positioning of the cards on the Talking Mats.

### 2.5 Procedure for Collecting, Transcribing and Analysing Data

Prior to completing the Talking Mats, participants were informed that there were no right or wrong answers and that it was how they felt that was important. Participants were also asked if they were happy for the interviews to be video recorded and participants facilitated the recording by setting up the recording equipment, stopping and starting recordings and taking screenshots of their completed Talking Mat. To reduce the power dynamics further, the interviews were conducted in a place of participants' choice and where they could easily leave and return to their daytime routines. Participants chatted to the interviewer before completing the Talking Mats to build rapport with the interviewer. It is also important that interviewers develop a non‐judgmental and sympathetic relationship (Anyan 2013) and therefore, checking placement of cards was used but without coercion or encouragement to consider changing their response.

There are legal and ethical implications related to the recording and dissemination of video and photographic materials collected for research (Balmer et al. 2015). Therefore, to protect confidentiality, only the research team had access to the recorded video material where the individual was identifiable. Consequently, to support interpretation and dissemination of the findings, participants' nonverbal communication needed to be transcribed alongside the verbal commentary.

The method used to analyse the different data formats was influenced by seminal qualitative researchers and perspectives including Onwuegbuzie and Byers (2014), Onwuegbuzie et al. (2010), Miles et al. (2020) and qualitative data analysis and notation systems used within conversation analysis. The influence of Sidnell and Stivers' (2013) conversation analysis was limited to the approach to completing notation as opposed to the tenets of conversation analysis, given that the aim of the study was not concerned with the structural underpinnings of language, nor the study of the social interactions captured within the video recordings.

The transcribing of nonverbal data was performed using a notation system influenced by Gorden's (1980) typology of nonverbal communication, which includes kinesics, proxemics, chronemics, and paralinguistics. During transcription, only nonverbal communications that were perceived by the transcriber as expressing content, symbolic or at least related to semantic content or feelings of the participant to some extent were transcribed. This excluded, for example, hand movements when they served only a functional purpose, such as taking and placing cards on the Talking Mat, unless accompanied by pauses or gestures that appeared to carry communicative meaning. Stereotypical movements of participants, such as fidgeting or adjusting their glasses, were also omitted to reduce the feeling of being cognitively overwhelmed during data analysis (Miles et al. 2020; Krauss et al. 1996). A sample of all the scripts was verified by a second researcher, and amendments were made following further review of the detail contained within transcripts.

To support transcription, notation systems that are commonly used within conversation analysis were reviewed (Sidnell and Stivers 2013). This enabled the researcher to create a notation system that could be adjusted during transcription in an iterative manner to enable participants' nonverbal and paraverbal communication to be captured (see Table 1). Notation was used in the transcription of the verbal prose to indicate where a change in intonation and hand or body movements occurred. The notation indicated the onset of the movement or change in pitch, volume, or tone and was attached to the word when only a single word was pronounced differently. To provide elaboration and clarification of the nonverbal data, a second column detailing idiosyncratic movements was then provided alongside the oral transcriptions.

**TABLE 1:** Notation system.

Notation symbol	Description
(1)	One second pause
(2)	Two second pause
(3)	Three second pause
(H)	Hesitation
(FG)	Facial gesture
(HM)	Head movement
(RH)	Right hand movement
(LH)	Left hand movement
(BH)	Movement with both hands
(UM)	Upper body movement
(WM)	Whole body movement
(>)	Increased volume
(<)	Decreased volume
(˄)	Increased pitch
(˅)	Decreased pitch
(IT)	Increased tone (happy tone)
(DT)	Decreased tone (sad/angry tone)
(P)	Proximity to objects changes
(PO)	Proximity to others changes
(L)	Laughs
(C)	Coughs
(S)	Sighs

After transcribing the interviews, the data sets were triangulated. Denham and Onwuegbuzie (2013) argue that triangulation of verbal and nonverbal communication affords qualitative researchers the opportunity to corroborate speech, to capture underlying messages, and develop insight. Therefore, the symbolic notation and descriptive explanations of the nonverbal communications were coded using NVivo software. Four pre‐defined codes were used (as per Table 2), based on the work of Denham and Onwuegbuzie (2013).

**TABLE 2:** Codes for triangulation of verbal and nonverbal data.

Code	Inclusion criteria	Exclusion criteria	Example
Corroborate	When nonverbal data and verbal converge, are aligned and are consistent with each other	Non‐convergence of verbal and nonverbal data	Head nods when saying ‘yes I feel…’
Capture	When the nonverbal data enhances or clarifies the verbal data	When the nonverbal data does not add additional information to portray the persons external or internal world	Moving hand in a claw shape towards head when talking about fuzzy and disturbed thoughts.
Discover	When nonverbal contradicts the verbal and needs further explanation or insight.	Convergence of verbal and non‐verbal data	Smiling when saying ‘yes, that is a problem’.
Broaden	When nonverbal communication widens the scope of understanding by expanding or developing new directions of thought or insight.	When nonverbal data does not provide insight about the interview, participants' understanding and abilities, and wider topics.	Hesitation when unclear about the question/answer.

## 3 Findings

Four talking mats were completed by six participants, either in one meeting or over two meetings. Participants spent between 25 and 102 min (excluding refreshment breaks) completing the four Talking Mats and providing additional information about their sleep. The interview length included participants talking about sleep concerns that were not covered in the Talking Mat activity but which they felt were important for the research. Questions were asked about how the person viewed, felt, or experienced aspects of their sleep, which were depicted in the picture cards.

Comparison of the transcribed verbal data with and without the nonverbal transcript is illustrated in a quote from participant 3. The quote reflects the content that was verbally expressed and shows how he described the environment, changes in time, and activities he was engaged in.

I have purchased some black curtains, put those up and it kills all the light. I have got those up in the bedroom window. So, I have had those shut sometimes…Sometimes sleep fine and another time was up at 7 o'clock one morning and still awake at 7 o'clock the following morning so up for the whole 24 hours and felt fine and was okay and then other times I will be watching telly, last of the summer wine, and then just switch telly off and go to bed. Switch the telly off, pull the curtains or put a sleep mask on and then go to sleep. Then maybe 2 o'clock wake up and feel could go to sleep for an hour and wake up feeling, really how long have I slept it must been 3–4 hours, what half an hour but feel really fit its weird.

The addition of the participant's nonverbal and paraverbal communication, such as yawns indicating how he feels tired, touching of the eyes indicating the impact of feeling tired on his body, hand gestures signalling how time moves on, changes to tone and pitch to emphasise feelings, shrugging of the body when saying it is ‘fine’ to suggest he feels it is unexplainable or confusing, and using nonverbal cues to indicate dozing off, added meaning to the verbal descriptions.

(sits back, rocks in chair) I have purchased some (covering eyes with arm) black curtains, put those up and it kills all the light (indicates with hand the light coming down). I have got those up in the (yawns) bedroom window. So, I have had those shut sometimes… Sometimes sleep fine and another time (rubs eyes) was up at 7 o'clock one morning and still (right hand points around an imaginary clock) awake at 7 o'clock the following morning so up for the whole 24 hours and felt (both shoulders come in and move forward) fine and was okay and then other times I will be watching telly last of the summer wine and then (head moves as though falling asleep) just switch telly off and go to bed. Switch the telly off, (mimes pulling curtains together) pull the curtains or put a (covers eyes as though holding mask) sleep mask on and then go to sleep. Then maybe 2 o'clock wake up and feel could go to sleep for an hour and wake up feeling, (increased pitch)really how long have I slept it must been 3‐4 hours, what half an hour but feel really fit its (increased pitch)weird.

Further examples have been illustrated to show the transcribing of verbal, nonverbal and paraverbal communication, in Table 3.

**TABLE 3:** Transcription of verbal and nonverbal communications for participants 1 and 3.

Verbal transcription	Nonverbal transcription
Participant 1. (BH) When I get off to sleep I think hypnagogia. It is a state where your (LH) body goes to sleep and your mind stays awake and what happens is, it does a number of different things to you. Sometimes you feel that you are being (BH2) pinned on the bed really fast, other times you feel that you are being (BH3) thrust around the room at a hundred miles an hour, really fast in circles. At other times you are being (BH4) thrust towards a wall or a door, so much so that you can even physically (BH5) see what is behind the door and then often what happens is your sometimes get these hallucination type things and when I have it is always darkness and there is nastiness and then sometimes I have like once I had these (BH6) creatures turn up that had these bright white eyes glaring at you, biting at my body and then I came out of it. And, I could feel the sensation in my body like it had happened. Then I had one, (LH2) not last week but the week before, and I told XXX and says this one I had I says I went into it, I didn't get the (BH7, 1) when things go fast but I knew it was a hypnagogia state because this black man (BH8) came up and said I am the black devil and have come to take your soul now. I managed to wake up out of it, (BH9) but I was freaked. Every time I have to go calm down, (LH3) relax, relax, calm down. Sprayed my pillow (LH4) with lavender spray, said (LH5) relax, calm down, relax and then eventually I can drop off to natural sleep. But, that can sometimes happen two or (BH10) three times at night that state and it is just a natural state so it doesn't, not going to harm your (BH11) health but it is not nice to be in it because it just always freaks me out. Now and again I can find if I am in it and it is where the (BH12) hallucinations I find if I can really focus I can make something nice happen and then the badness (BH13) seems to then disappear but often it is only when you are in that state that something so negative about it, it is never nice, it is not like when you go to sleep and you have a nice dream or one of those umm lucid dreams where you can (LH6) control. They are nice cos you can control them and make nice (LH7) things happen but this is always, the state is always, I have to shake myself out of it I can never go to sleep in it as sleep in (BH14) that state as never have a good night.	Sat arms folded hood of jumper up BH‐ Hands interlinked in front LH Claws over body then out from mind BH2 Pin hands down BH3 Circles hands large circles going forward BH4 hands fly out and to right BH5 points near eyes and then points to outside where door could be BH6 Creatures—using pinched finger over eyes then Imitates biting body LH2 beating with finger as pointing BH7 hands circling BH8 hands fold across chest BH9 hands shake held out in front LH3 hand over chest beating with heart LH4 mime spray LH5 Relax—hands moving down body BH10 hands pointing in opposite directions back and forth BH11 hand in claw position fingers down and bounces forward BH12 hands move forward and shaking about BH13 then shoot off (disappear) LH6 hand making small circles LH7 Hands start to circle in upward motion BH14 claw hands coming in towards each other (pushing/locked in)
Participant 4. (FG) I could (RH) sleep for England, like this morning I got up to take the dog out by 12 o'clock (RH2) I wanted to go to bed for a couple of hours, (FG) but still couldn't sleep much… Before (RH3) XXX died I could sleep for anything. Now at the weekends I wake up at the same time at 8 o'clock. I don't know (RH4) why I cant lie in, (HM, FG) its weird. At weekend I used to be able to sleep till 10 or 11, 12 o'clock, now 7 or 8. At 12 I am tired again hoping to go back to bed	FG smiles RH pointing using beats RH2 pointing backwards over shoulder FG smiles RH3 open hand raised with hand flat then hand beating when talking RH4 hand circles then opens up HM shakes head, pulls lips downwards in sad face

The excerpts presented in Table 3 show how, within the example of participant 1, the nonverbal communication portrayed thoughts and ideas through using hand movements to create an imaginary or abstract object within the space between the participant and the interviewer. McNeill's (1992) classification identifies this as metaphoric nonverbal communication. In contrast, gestures that stimulate the movement of objects are labelled as iconics, depicted in the example of participant 3 with the closing of the curtains. Furthermore, participants used beats through up and down movements of the hands to indicate timing or to show the interviewer when they were making important points which they felt needed to be accentuated or acknowledged. In addition, emblems such as head nods and shakes, which are gestures with specific linguistic designations that have shared meaning across the participants and the interviewer, were recorded and transcribed for analysis. Finally, participant 4 shows the use of deictics to indicate time that has passed (see Table 3), and this was also seen in the transcripts to indicate time that is yet to happen by pointing forward to the future.

In addition to the McNeilll's classification system, nonverbal communication also supported emotional expression. Intonation and facial gestures were often used to express emotions, particularly like or dislike of an object or idea, as well as to express apprehension or dissatisfaction regarding anticipated future events as illustrated in quotes by participant 2, 5, and participant 6.

Participant 2: Talking about sleep apnoea.

I (shakes head) don't understand what it is. No, the doctor just says that I suffer from it and that I have to go back and see him. I hope that I don't suffer from it in the middle of me sleep. (decreased tone) I do snore quite a lot though. My husband snores but he is like, I don't (increased volume)copy him but if I am dozing off, I snore a bit and then quickly waking up again.

Participant 5: Talking about needing to get up in the night to use the toilet.

(Screws up nose) Normally, I drink out the tap, just water… cos I have to get up all the time. Do you think it would make it easy (hand moves from in front to the side of the table, then bouncing back and forth in beats) if I drink less water. If I drink less water, then I don't really have (1 second pause) yes.

Participant 6: Talking about waking in the night.

I wake up in the night, getting food and that (slight smile)

Interviewer: Is that because you are hungry?

Yes, I get my cheeses and that. (smiles) Dairy cheeses.

As anticipated, the majority of nonverbal communication simply corroborated the verbal speech and these included transcriptions of head nods, shakes and hand gestures to indicate how things can change from one day to the next. The latter was indicated through positioning of the hands over the Talking Mat and moving back and forth from the side of the mat with the picture of a ‘thumbs up’ to the other side of the mat with a picture of the ‘thumbs down’, before settling on the centre column to indicate ‘so‐so’, or through rotating of the hand to show how it can alternate between being a problem and not being a problem. In addition, indicating changes in time was communicated nonverbally through pointing forwards, backwards or using beats of the hand to signal how, after a period of time, the next step was taken. Hours or days of the week were also visually illustrated through counting on fingers or pointing to the time on an imaginary clockface.

The actions of others and identification of where objects are in space in relation to the narrative were coded as ‘corroborate’ as well as ‘capture’. These could also indicate internal feelings and location on the body where these were felt, such as touching the head to indicate confusion or changes in thought processes.

Intent and emotional state were captured through both paraverbal and nonverbal communication with intonation, pitch, and volume, which were used to emphasise words associated with sadness, anger, uncertainty, surprise, really liking something, or happiness. Elaborating feelings within statements could easily be missed if only the verbal speech was transcribed and allowed the participant to express feelings without needing to find the words to explain. Participants were also able to use nonverbal communication to set the scene so that additional words and explanations were no longer needed. For example, to explain textures and firmness of bedding and when recalling actions or using different pitches to indicate comments made by other people.

The ‘discover’ code highlighted transcripts where nonverbal communication was incongruent or did not directly correlate with the expressed speech. It is unclear whether these brief moments were a result of self‐denial, indecision, or trying to reach clarity to provide an answer. One nonverbal utterance that was coded as ‘discover’ included a participant's hand movements going forward as though talking about the future despite talking about the death of a loved one. This inconsistency could indicate a variety of meanings, such as that they perceive the person as very much still in their life and their memories always being part of their future as opposed to a lack of acceptance. Thus, caution is needed when interpreting nonverbal communication due to the subjective nature of interpreting nonverbal modes of expression. Discrepancies between verbal and nonverbal data could not always be resolved. However, reflecting on what was said previously or later in interviews could give the researcher some insight into why incongruence between verbal and nonverbal communication was recorded. For example, one participant explained that feeling tired in the day was a problem for her while smiling and nodding; however, previous and later discussion inferred that the smile was relating to the feeling that exactly the right thing had been said to uncover the issue. In other words, there could be multiple layers of meaning going on at the one time. Therefore, as interpreters of nonverbal communication, researchers may not always be aware of all of the meaning, but reflections on why inconsistency occurs can provide additional insight and meaning. This reflection needs to be completed following researchers’ immersion in the participants’ complete data set.

Two broader issues identified from the nonverbal communication included the extra time needed for the research participants to process questions and come to a decision, and the problem with recalling the overall question. This was evident through their hesitation and pauses before providing an answer and through their questions asking for clarification of the correct pile to place the cards beneath.

Some nonverbal communication captured in the videos highlighted a broader issue of participants interacting with the environment around them. This included interacting with staff, commenting on noises from other rooms, and interacting with the technology being used. For one participant, nonverbal interactions with the computer screen in which they could see themselves being filmed included eye gaze, smiling, doing a thumbs up gesture, and looking directly into the camera. For a few participants, it became apparent during the interview that they preferred to have control over the filming and quickly took on the role of starting and pressing the button to end the recording as well as being active in taking screenshots of the Talking Mat. This enabled participants to control when interviews should be paused as well as the positioning of the camera. This was identified and coded as part of a broader consideration about positioning participants as equal partners and reducing perceived or imposed role hierarchies.

Challenges emerged during the transcribing and coding of nonverbal data, including differences across participants' nonverbal expressiveness. Differences in nonverbal and paraverbal expressiveness were perceived as related to individual differences, neurodiversity, links to medication and medication changes. Some participants were more elaborate and explicit in their nonverbal communications while others used smaller movements and had reduced facial expressions. The latter required additional time and re‐reviewing of video footage due to difficulties capturing their verbal and paraverbal communications. Reflexivity was important when transcribing nonverbal data and was supported by a second member of the research team who reviewed excerpts of transcriptions alongside the video footage.

## 4 Discussion

Hayden et al. (2024) highlight that observations of nonverbal communication enable researchers to look for matches and mismatches with the verbal communication, and this paper extends this method to systematically analyse the verbal and nonverbal communication through triangulation to gain broader insight and depth of meaning. Within the study reported, the cross‐case analysis of the nonverbal communication of participants when transcribed and triangulated with the verbal speech showed corroboration and enhanced, clarified, and elaborated the spoken narratives. More specifically, the depth came from the nonverbal communication enriching the ability of the interviewer to understand the emotions and to support a mental imagery to unfold which can be coined as setting the scene. Interpreting only verbal communication could therefore result in a loss of information that the participants consider as important: their attitudes, emotions and feelings.

The participants used nonverbal communication, such as head nods, shakes, smiles or grimaces, to reinforce and show strength of their attitude, belief or perception. Attitudinal data ranged from wide smiles and increased tone to indicate pleasure from an experience to a decreased tone portraying annoyance at other people for not believing what is being recalled. In addition, attitudinal expression of disagreement with others was communicated through hand movements that swept diagonally, as if dismissing or motioning away others' comments. These nonverbal and paraverbal communications can be easily lost in verbal transcription; however, paying attention to these modes of expression can enable the researcher to have a better grasp of the meaning of the data (Point and Baruch 2023), providing a more comprehensive picture during analysis. Nonverbal communication indicated size, shape, position, and temporal aspects of abstract and concrete phenomena through hand and body movements. This was observed when participants described time, places, and previous actions. Hand gestures indicated where objects were in relation to themselves as well as the speed of movements or that time was elapsing, as illustrated by describing a sense of spinning elaborated by the rapid velocity of hands circling, or slow movements indicating a prolonged transition of time or change in perspective. The enactment or nonverbal presentation of the experience provides insights as to how the participant understood and interpreted the experience. This meant that participants were not required to find additional words in which to describe and explain these experiences but could freely tell their stories. This is particularly important when interviewing people with cognitive disabilities who may struggle to find the exact words to explain past experiences. Information not related to the phenomena of sleep problems was also communicated nonverbally rather than verbally. Nonverbal communications such as pauses and hesitations longer than the participant's typical response time indicated when they were unsure of what they had been asked, and folded arms could be seen as a closed posture that could be an indication of not wanting to pursue a topic of conversation (Foley and Gentile 2010). Therefore, paying attention to nonverbal communication alongside verbal communication was perceived as adding depth, clarity, and reducing misunderstandings between the interviewer and the participant, though all communication remains uncertain and open to interpretation.

Nonverbal communication is often unconscious and can often offer an accurate depiction of a person's feelings and therefore can support researchers to reflect on signs of social acquiescence within interviews. Kazdin (2014) suggests that the aim of qualitative research is to obtain credible findings which capture the participants' experiences. Analysis of nonverbal communication alongside the spoken word afforded the researcher greater feelings of confidence in the interpretation and conclusions drawn, in that findings were perceived to be more plausible and trustworthy and superior in capturing the participants' lived experiences.

### 4.1 Strengths and Limitations

A strength of the research is that it supported people with intellectual disabilities who are verbal to share their feelings and lived experiences as part of qualitative research, which could be applied to practice settings. Research that amplifies participants' agency and voice guided by principles of mutual respect promotes inclusivity for populations and enables those supporting them to gain insight and understanding about their lives and internal world. The approach used could be replicated across research questions that explore the experiences, feelings, and beliefs of people with intellectual disabilities who are verbal communicators. For practitioners who support people with intellectual disability, the use of video recording and taking note of nonverbal as well as verbal communication could offer affirmability following use of communication tools such as Talking Mats to support person‐centred care planning and decision making. During this process, practitioners should observe for where verbal and nonverbal communications corroborate, capture, or discover additional insight about a person's feelings, beliefs, or experience. Furthermore, nonverbal communications can broaden the practitioner's knowledge to enable them to reflect on their interviewing technique and reflect on the individual with intellectual disabilities' ability and understanding regarding the task.

Taking time to build rapport with participants and comprehensive transcription and analysis of nonverbal communications can aid understanding and interpretation of data. However, knowing the person well has not been found to increase inter‐rater reliability when inferring meaning from nonverbal communication during qualitative research (Pearlman and Michaels 2019). Certainly, transcribing and analysing nonverbal communication was perceived by the researcher as more subjective and open to interpretation compared to analysing the verbal data. Nind (2008) agrees that interpreting nonverbal communications is difficult and should be done with caution. Therefore, checking findings is important to ensure confirmability. Furthermore, Fusch et al. (2018) advocate that methodological triangulation that relies on within‐method research still has the flaws inherent in the one method used and therefore, these flaws impact both data sets or types of data.

Manual transcription carries the risk of errors, especially when the task becomes long and arduous (Point and Baruch 2023). Errors and bias need mentioning because the time to record nonverbal communication was greater than the time taken to transcribe verbal communication and this task was open to interpretation of the transcriber. Not every eye movement, glance, change in posture, tone, or change in speaking rate was transcribed; therefore, it was down to the transcriber to notice and identify the nonverbal communication. Following transcribing the verbal data, the video recordings required viewing and reviewing to pay attention to nonverbal communication in an iterative way, where new observations led to further attention being paid to micro communications. Although this can be seen as subjective, J. Van Manen (1979) argues that interpretation of data is always incomplete and should not be treated as fact, while Foley and Gentile (2010) ask the pertinent question about ‘when should you stop transcribing?’. Therefore, a decision was made about the depth or density of the transcription to enhance the credibility versus the risk of losing sight of what is important for the research question.

A further limitation of this research is that analysis of the verbal data that excludes nonverbal elements was not conducted to check for differences in interpretation and substantiate the claims made. However, a secondary analysis was not possible for this study given the familiarity with the nonverbal communications that was gained from reading and re‐reading these transcripts. It is proposed that future research studies conduct separate analysis of the transcripts with and without the nonverbal transcripts to highlight if the interpreted meaning and depth of insight changes when including the transcribed nonverbal interactions.

The use of video equipment, which was noticeable within the interviews, could negatively impact participants' level of comfort, the interviewer‐interviewee relationship and the dialogue. Allowing participants' control over starting and stopping recordings, changing the angle of the camera and not having interviews recorded enabled participants to feel more at ease during the interviews. One participant requested that part of their interview was not recorded and dictated what the interviewer should write. This flexibility in the approach ensured that participants felt comfortable discussing potentially embarrassing or personally sensitive issues and provided an opportunity for them to have input on what was recorded after they had time to consider it and reflected upon how they wanted the information to be portrayed. Flexibility in approach contributes to establishing respect for research participants, which is indeed paramount.

### 4.2 Further Research

Advancing qualitative interview methods in research relating to people with intellectual disability, dementia and stroke is needed (Nocivelli et al. 2023; Caldwell 2014), as researchers increasingly seek to amplify the voices and perspectives of participants with communication and intellectual disabilities (Charalambous et al. 2022; Dreyfus 2022). When conducting research with people with intellectual disabilities, it is important to consider what data to collect and how.

Researchers should carefully consider further ethical implications of video recording participants. Participants within this study were informed about who would have access to the videos and how these would be transcribed to remove identifiable information. Participants indicated on consent forms that they understood that no videos, still images, or names would be included in the dissemination of findings. This was particularly important to one participant and their family who sought further reassurances from the interviewer that the videos would not be placed in the public domain.

Given the advancements in software available to support data analysis, time required to transcribe nonverbal and verbal communication, and the risk of error or missing data, it is conceivable that directly coding segments of the videos is a viable and preferred option. However, caution needs to be taken with the sharing of these videos to protect participant confidentiality. Therefore, transcription of the nonverbal and paraverbal communications would still be recommended to support dissemination of data.

Further research is needed, given that the study reported here only included six participants using Talking Mats as a communication tool. It is put forward that research that looks at transcribing and analysing participants who have an intellectual disability and who use a range of alternative and augmentative systems to communicate their thoughts, preferences and needs is warranted. It is envisioned that people who use low and high technology communication systems or researchers who select a range of communication tools to support data collection would benefit from transcribing and analysing nonverbal interactions. Therefore, a call is put forward for other researchers working across a variety of disciplines, a range of topics, age groups and cultures to gauge the effectiveness and to identify the merit and boundaries of triangulating and interpreting nonverbal data alongside transcripts of the spoken word.

## 5 Conclusion

It is important that practitioners and researchers adopt an approach that enables participants with intellectual disabilities to articulate their experiences on their own terms. Innovative ways of conducting qualitative research with people who have intellectual disabilities to provide the opportunity for people to express their lived experience are needed. This article has set out an innovative way to transcribe and analyse nonverbal data as part of qualitative research that relies on interviewing. This has benefits for people with intellectual, cognitive and communication disabilities who may rely on expressing their feelings and emphasising what is most important to them through intonation, body movements and facial gestures. It is also beneficial for the wider community of researchers, clinicians and those with an interest in the topic as it provides depth to insight and greater understanding into the perspectives of people they support.

## Funding

The authors have nothing to report.

## Conflicts of Interest

The authors declare no conflicts of interest.

## Data Availability

The data that support the findings of this study are available on request from the corresponding author. The data are not publicly available due to privacy or ethical restrictions.
